# Association of Mechanical Ventilation and Flue Use in Heaters With Asthma Symptoms in Japanese Schoolchildren: A Cross-Sectional Study in Sapporo, Japan

**DOI:** 10.2188/jea.JE20130135

**Published:** 2014-05-05

**Authors:** Shi Cong, Atsuko Araki, Shigekazu Ukawa, Yu Ait Bamai, Shuji Tajima, Ayako Kanazawa, Motoyuki Yuasa, Akiko Tamakoshi, Reiko Kishi

**Affiliations:** 1 Hokkaido University Graduate School of Medicine, Department of Public Health Sciences, Sapporo, Japan; 1 北海道大学大学院医学研究科公衆衛生学分野; 2 Hokkaido University Center for Environmental and Health Sciences, Sapporo, Japan; 2 北海道大学環境健康科学研究教育センター

**Keywords:** asthma, heating, mechanical ventilation, child, indoor air quality

## Abstract

**Background:**

Use of fuel heaters is associated with childhood asthma. However, no studies have evaluated the associations of flue use and mechanical ventilation (ventilation) with asthma symptoms in schoolchildren.

**Methods:**

This cross-sectional study investigated schoolchildren in grades 1 through 6 (age 6–12 years) in Sapporo, Japan. From November 2008 through January 2009, parents completed questionnaires regarding their home environment and their children’s asthma symptoms.

**Results:**

In total, 4445 (69.5%) parents of 6393 children returned the questionnaire. After excluding incomplete responses, data on 3874 children (60.6%) were analyzed. The prevalence of current asthma symptoms and ever asthma symptoms were 12.8% and 30.9%, respectively. As compared with electric heaters, current asthma symptoms was associated with use of flued heaters without ventilation (OR = 1.62; 95% CI, 1.03–2.64) and unflued heaters with ventilation (OR = 1.77; 95% CI, 1.09–2.95) or without ventilation (OR = 2.23; 95% CI, 1.31–3.85). Regardless of dampness, unflued heaters were significantly associated with current asthma symptoms in the presence and absence of ventilation.

**Conclusions:**

Use of unflued heaters was associated with current asthma symptoms, regardless of dampness. In particular, the prevalence of current asthma symptoms was higher in the absence of ventilation than in the presence of ventilation. Ever asthma symptoms was only associated with use of unflued heaters without ventilation. Consequently, use of fuel heaters, especially those that have no flue or ventilation, deserves attention, as their use might be associated with childhood asthma symptoms.

## Abstract (ja)

**【背景】:**

暖房器具の種類と児童の喘息との関連が報告されている。しかし、これまでに児童の喘息と暖房器具の排気管、および機械換気との関連を評価した研究はなかった。

**【方法】:**

札幌市で小学校１年生から6年生（6−12歳）を対象に横断研究を行った。2008年11月から2009年1月にかけて、保護者に住宅環境および児童の喘息症状に関する調査票の記入を依頼した。

**【結果】:**

6393名の児童のうち、4445人から調査票を回収し（回収率69.5%）、欠損データを除外した3874人のデータを分析した。現在喘息症状がある児童は12.8%、今までに喘息症状がある児童は30.9%であった。電気暖房と比較し、ガス、石油などの暖房で排気管あり・機換換気なしの場合（OR = 1.62; 95% CI, 1.03-2.64）、排気管なし・機械換気ありの場合（OR = 1.77; 95% CI, 1.09-2.95）、排気管なし・機械換気なしの場合（OR = 2.23; 95% CI, 1.31 -3.85）は、いずれも現在の喘息症状のオッズ比が有意に高かった。ダンプネスの有無に関わらず、排気管なしの暖房を利用している場合は機械換気がある場合とない場合のいずれも現在の喘息症状と有意に関連していた。

**【結論】:**

ダンプネスがあってもなくても、排気管なしの暖房器具を使うことは現在の喘息症状と関連していた。特に、機械換気がある場合と比べ、機械換気がない場合は現在の喘息症状のオッズ比が有意に高かった。一方、今までに喘息症状があることは、排気管がない暖房器具を機械換気なしで使用する場合とのみ関連していた。したがって、ガス、石油等の暖房器具に排気管が取り付けられない場合、および機械換気がない場合には、児童の喘息に関連する可能性があることに十分注意することが必要である。

## INTRODUCTION

Asthma is characterized by recurrent attacks of breathlessness and wheezing and is a common chronic disease among children.^1^ Asthma is associated with a significant socioeconomic burden, as it imposes high health care costs on households^2^ and decreases school attendance.^2^^,^^3^ Among schoolchildren aged 6 to 14 years, the prevalence of asthma ranges from 10.5% to 18.2%^4^ and has been steadily increasing in Japan.^5^^,^^6^

Epidemiologic evidence suggests that environmental factors are major risk factors for asthma in children.^7^ Tobacco smoke,^8^ carpeting,^9^ living near main roads,^10^ and indoor dampness and mold^11^^–^^13^ all cause or exacerbate asthma and allergic symptoms in schoolchildren. In particular, indoor dampness is a strong risk factor for asthma.

It has been suggested that indoor smoke from combustion of solid fuels such as coal and biomass leads to the development of asthma and other respiratory symptoms in children.^14^ The World Health Organization states that over 3 billion people in developing countries depend on solid fuel for heating and cooking; however, Japanese households do not use solid fuels.^15^

Numerous studies have reported that use of fume-emitting fuel heating in the home can adversely affect childhood asthma symptoms. The fuels used include wood,^16^ natural gas (gas),^17^ and kerosene.^18^ However, a cross-sectional study reported that fume-emitting heaters were not associated with asthma symptoms, as compared with non–fume-emitting heaters.^19^ Three interventional studies suggested that installation of a flue on unflued heaters decreased asthma symptoms.^20^^–^^22^ In addition, mechanical ventilation (ventilation) is a useful method of decreasing indoor air pollution.^23^ However, it remains unclear whether ventilation systems decrease the effects of heating systems on asthma symptoms.

In Sapporo city, Japan, fuel heaters are commonly used during winter to heat the home, but the association between the use of such heating systems and asthma symptoms remains unknown. We investigated the association of the combination of ventilation and flue use in heaters with asthma symptoms among Sapporo schoolchildren who were exposed to fuel heaters in poorly ventilated houses during winter.

## METHODS

### Study participants

This cross-sectional study was conducted in public elementary schools in Sapporo, Japan. The details were described in our previous study.^24^ In brief, 12 public schools in Sapporo agreed to participate in the study. Between November 29, 2008 and January 30, 2009, a total of 6393 children in grades 1 to 6 (age 6–12 years) received a questionnaire from their teachers, which included instructions for their parents to complete the questionnaires. Classroom teachers collected the questionnaires after they had been completed.

### Definition of asthma symptoms

Asthma symptoms were defined using the Japanese Version^25^ of the International Study of Asthma and Allergies in Childhood (ISAAC) questionnaire.^26^ We classified participants as having “current asthma symptoms” when they answered “Yes” to the question, “Has your child had wheezing or whistling in the chest in the last 12 months?”. “Ever asthma symptoms” was defined as an affirmative response to the question, “Has your child ever had wheezing or whistling in the chest at any time in the past?”.

### Assessment of heating systems and mechanical ventilation status

We assessed heating systems and mechanical ventilation by asking 3 questions on the use of heating fuels, heater flue use, and ventilation status.

Regarding heating fuels used, we asked: “What heating fuels do you use in your home?”. Participants could choose 1 or more of the following: kerosene, gas, electricity, or others. According to the answers, heating fuel was redefined as follows: electricity—use of only an electric heater in the home; kerosene—use of any type of kerosene heater; gas—use of any type of gas heater but not a kerosene heater; others—use of other types of fuel heaters only or using other fuels in combination with an electric heater.

Regarding heater flue we asked: “Are you using unflued heaters that have no vent pipe to the outside?”. Participants could answer “Yes” or “No”. Next, heating fuels and heater flue status were combined and classified as electric heaters, flued heaters (not including electric heaters), and unflued heaters (not including electric heaters).

Ventilation status was assessed by the question, “Which rooms in your home have mechanical ventilation?”, and the possible answers were “living room”, “children’s bedroom”, “kitchen”, “bathroom”, “toilet”, and “other sites”. In the analysis, ventilation status in the living room and/or children’s bedroom was used to define presence of mechanical ventilation (ventilation). Finally, the status of heating system and ventilation was classified into 5 groups: electric heaters (only), flued heaters with ventilation, flued heaters without ventilation, unflued heaters with ventilation, and unflued heaters without ventilation.

### Assessments of other aspects of the home environment

Other parameters were also evaluated in the questionnaires, namely, residence within 200 meters of a main road (yes/no), presence of wall-to-wall carpeting in the home (yes/no), presence of furry animals and/or birds in the home (yes/no), presence of a smoker in the home (yes/no), presence of visible mold (yes/no), perception of moldy odor (yes/no), presence of water leakage within the past 5 years (yes/no), and condensation on windowpanes (yes/no). Additional information was also collected, including sex, age (6–12), school grade (1–6), whether the child was first-born, and parental history of allergies (both parents, mother only, father only, or neither).

### Statistical analysis

Categorical variables are presented as number of children (percentage). The χ^2^ test was used to analyze associations of asthma symptoms with participant characteristics and home environment. Logistic regression analyses were used to calculate odds ratios (ORs) and 95% confidence intervals (95% CIs) for the effects of home heating systems and ventilation on asthma symptoms. Electric heaters were used as the reference and categorized as non–fume-emitting heaters, regardless of ventilation status. All analyses were performed using JMP version 10.0 for Windows (SAS Institute Inc., Cary, NC, USA). Statistical significance was defined as a 2-sided *P* value of less than 0.05.

We controlled extensively for potential confounders in the multivariate models. We chose these confounders on the basis of the results of our study (ie, those significantly associated with asthma symptoms) and risk factors reported in previous studies.^8^^–^^13^ In model 1, we adjusted for children’s characteristics such as sex, school grade, school, and parental history of allergies. In model 2, we adjusted for home environmental factors such as residence within 200 meters of a main road, presence of wall-to-wall carpeting in the home, and presence of a smoker in the home, in addition to model 1 covariates. In model 3, we adjusted for indoor dampness, in addition to model 2 covariates. Because indoor dampness is a strong risk factor for asthma, we conducted a stratified analysis according to indoor dampness after adjusting for the covariates in model 2.^11^^–^^13^ We used 4 dampness indicators to assess indoor dampness status, including visible mold, perception of moldy odor, water leakage within the past 5 years, and condensation on windowpanes. We defined absence of indoor dampness as the absence of all 4 dampness indicators in the home and presence of indoor dampness as at least 1 of the 4 dampness indicators in the home.

### Ethical considerations

This study was approved by the ethical board for epidemiologic studies at the Hokkaido University Graduate School of Medicine and conformed to the principles outlined in the Declaration of Helsinki of 1975, as revised in 1983. The study was conducted after obtaining relevant informed consent from the participants’ parents.

## RESULTS

In total, the parents of 4445 (69.5%) children replied to the questionnaires. After excluding questionnaires with incomplete data on sex, school grade, parental history of allergies, use of heating fuels, heater flue, ventilation, and asthma symptoms, data from 3874 (60.6%) children were evaluated, of which 496 and 1197 reported current asthma symptoms and ever asthma symptoms, respectively. These values correspond to prevalence of 12.8% and 30.9%, respectively.

Table 1 compares participants with and without asthma symptoms. Current asthma symptoms and ever asthma symptoms were more frequent in boys and in children with a parental history of allergies. The prevalence of current asthma symptoms was 15.6% in boys and 10.1% in girls, and the prevalence of ever asthma symptoms was 35.8% in boys and 26.1% in girls. The distribution of the prevalence of current asthma symptoms differed by school grade (13.4%, 14.0%, 16.0%, 10.4%, 13.2%, and 9.7% for grades 1–6, respectively). In the 12 schools, the prevalence of current asthma symptoms and ever asthma symptoms varied from 6.6% to 15.3% and from 21.8% to 36.0%, respectively.

**Table 1.** Associations between asthma symptoms and participant characteristics

Variable	*n* (%)	Current asthma symptoms		*P*-value	Ever asthma symptoms		*P*-value
	
		Yes *n* (%)	No *n* (%)		Yes *n* (%)	No *n* (%)	
Participants		496 (12.8)	3378 (87.2)		1197 (30.9)	2677 (69.1)	
Sex							
Male	1900 (49.0)	297 (15.6)	1603 (84.4)	<0.001	681 (35.8)	1219 (64.2)	<0.001
Female	1974 (51.0)	199 (10.1)	1775 (89.9)		516 (26.1)	1458 (73.9)	
School grade							
1st	635 (16.4)	85 (13.4)	550 (86.6)	0.006	202 (31.8)	433 (68.2)	0.59
2nd	656 (16.9)	92 (14.0)	564 (86.0)		196 (29.9)	460 (70.1)	
3rd	655 (16.9)	105 (16.0)	550 (84.0)		204 (31.2)	451 (68.8)	
4th	689 (17.8)	72 (10.4)	617 (89.6)		224 (32.5)	465 (67.5)	
5th	612 (15.8)	81 (13.2)	531 (86.8)		194 (31.7)	418 (68.3)	
6th	627 (16.2)	61 (9.7)	566 (90.3)		177 (28.2)	450 (71.8)	
Parental history of allergies							
Both parents	994 (25.7)	197 (19.8)	797 (80.2)	<0.001	432 (43.5)	562 (56.5)	<0.001
Mother only	946 (24.4)	158 (16.7)	788 (83.3)		343 (36.3)	603 (63.7)	
Father only	531 (13.7)	69 (13.0)	462 (87.0)		184 (34.6)	347 (65.4)	
Neither parent	1403 (36.2)	72 (5.1)	1331 (94.9)		238 (17.0)	1165 (83.0)	
First-born child							
Yes	2074 (53.5)	269 (13.0)	1805 (87.0)	0.512	669 (32.3)	1405 (67.7)	0.072
No	1759 (45.4)	224 (12.7)	1535 (87.3)		520 (29.6)	1239 (70.4)	
School							
1	462 (11.9)	64 (13.8)	398 (86.2)	0.049	141 (30.5)	321 (69.5)	<0.001
2	173 (4.5)	24 (13.9)	149 (86.1)		57 (33.0)	116 (67.0)	
3	519 (13.4)	73 (14.1)	446 (85.9)		180 (34.7)	339 (65.3)	
4	177 (4.6)	23 (13.0)	154 (87.0)		49 (27.7)	128 (72.3)	
5	209 (5.4)	32 (15.3)	177 (84.7)		74 (35.4)	135 (64.6)	
6	333 (8.6)	36 (10.8)	297 (89.2)		78 (23.4)	255 (76.6)	
7	364 (9.4)	39 (10.7)	325 (89.3)		124 (34.1)	240 (65.9)	
8	81 (2.1)	9 (11.1)	72 (88.9)		18 (22.2)	63 (77.8)	
9	441 (11.4)	67 (15.2)	374 (84.8)		146 (33.1)	295 (66.9)	
10	413 (10.6)	49 (11.9)	364 (88.1)		116 (28.1)	297 (71.9)	
11	431 (11.1)	62 (14.4)	369 (85.6)		155 (36.0)	276 (64.0)	
12	271 (7.0)	18 (6.6)	253 (93.4)		59 (21.8)	212 (78.2)	

Values are expressed as number (percentage). *P* values were calculated using the χ^2^ test.

Missing data are not displayed.

The associations of asthma symptoms with home environment are shown in Table 2. The variables associated with current asthma symptoms in children were use of kerosene and gas heaters, use of unflued heaters, absence of ventilation in the living room or bedroom, presence of wall-to-wall carpeting, presence of a smoker in the home, visible mold, moldy odor, water leakage, and condensation on windowpanes. The variables associated with ever asthma symptoms were absence of ventilation in the living room or bedroom, residence within 200 meters of a main road, presence of wall-to-wall carpeting, presence of a smoker in the home, water leakage, and condensation on windowpanes.

**Table 2.** Associations between asthma symptoms and the home environment

Variable	Current asthma symptoms		*P*-value	Ever asthma symptoms		*P*-value
	
	Yes *n* (%)	No *n* (%)		Yes *n* (%)	No *n* (%)	
Use of heating fuels						
Electricity	27 (8.2)	304 (91.8)	0.045	91 (27.5)	240 (72.5)	0.481
Natural gas	74 (12.7)	510 (87.3)		187 (32.0)	397 (68.0)	
Kerosene	379 (13.4)	2456 (86.6)		883 (31.2)	1952 (68.8)	
Others	16 (12.9)	108 (87.1)		36 (29.0)	88 (71.0)	
Flue status						
Flued	361 (12.0)	2659 (88.0)	0.004	916 (30.3)	2104 (69.7)	0.153
Unflued	135 (15.8)	719 (84.2)		281 (32.9)	573 (67.1)	
Ventilation						
Yes	302 (11.9)	2231 (88.1)	0.025	755 (29.8)	1778 (70.2)	0.044
No	194 (14.5)	1147 (85.5)		442 (33.0)	899 (67.0)	
Residence within 200 meters of a main road						
Yes	397 (13.5)	2545 (86.5)	0.057	954 (32.4)	1988 (67.6)	0.001
No	95 (10.5)	808 (89.5)		236 (26.1)	667 (73.9)	
Wall-to-wall carpeting in the home						
Yes	318 (14.4)	1885 (85.6)	0.002	720 (32.7)	1483 (67.3)	0.022
No	177 (10.7)	1480 (89.3)		473 (28.6)	1184 (71.4)	
Furry animals or birds in the home						
Yes	115 (11.9)	853 (88.1)	0.458	285 (29.4)	683 (70.6)	0.452
No	381 (13.1)	2523 (86.9)		911 (31.4)	1993 (68.6)	
A smoker in the home						
Yes	269 (14.6)	1576 (85.4)	0.004	608 (33.0)	1237 (67.0)	0.008
No	227 (11.2)	1798 (88.8)		589 (29.1)	1436 (70.9)	
Visible mold						
Yes	198 (14.3)	1187 (85.7)	0.015	444 (32.1)	941 (67.9)	0.397
No	295 (11.9)	2187 (88.1)		750 (30.2)	1732 (69.8)	
Moldy odor						
Yes	49 (23.6)	159 (76.4)	<0.001	79 (38.0)	129 (62.0)	0.076
No	447 (12.2)	3211 (87.8)		1115 (30.5)	2543 (69.5)	
Water leakage within the past 5 years						
Yes	85 (19.2)	357 (80.8)	<0.001	165 (37.3)	277 (62.7)	0.003
No	408 (11.9)	3012 (88.1)		1026 (30.0)	2394 (70.0)	
Condensation on windowpanes						
Yes	305 (15.0)	1726 (85.0)	<0.001	672 (33.1)	1359 (66.9)	0.004
No	189 (10.3)	1646 (89.7)		521 (28.4)	1314 (71.6)	
Indoor dampness						
Absent	158 (10.1)	1401 (89.9)	<0.001	443 (28.4)	1116 (71.6)	<0.001
Present	338 (14.6)	1977 (85.4)		754 (32.6)	1561 (67.4)	

Values are expressed as number (percentage). *P* values were calculated using the χ^2^ test.

Ventilation means mechanical ventilation in the living room or bedroom.

Missing data are not displayed.

With use of electric heaters as the reference, use of gas and kerosene heaters was associated with current asthma symptoms (Table 3). Furthermore, use of unflued heaters was associated with current asthma symptoms as compared with use of flued heaters, as was absence of ventilation versus presence of ventilation. After adjusting for potential confounders, including sex, school grade, school, parental history of allergies, residence within 200 meters of a main road, presence of wall-to-wall carpeting in the home, presence of a smoker in the home, and indoor dampness in model 3, use of kerosene heaters (OR = 1.58; 95% CI, 1.03–2.49) and unflued heaters (OR = 1.29; 95% CI, 1.02–1.63) remained significantly associated with current asthma symptoms, as compared with use of electric heaters and use of flued heaters. No significant association was found after adjustment for ever asthma symptoms.

**Table 3.** Multivariate analyses of the association between heating systems and mechanical ventilation (ventilation) and asthma symptoms

Variable	*n* (%)	Asthma symptoms *n* (%)	Crude OR (95% CI)	Model 1 OR^a^ (95% CI)	Model 2 OR^b^ (95% CI)	Model 3 OR^c^ (95% CI)
Current asthma symptoms						
Use of heating fuels						
Electricity	331 (8.5)	27 (8.2)	1.00	1.00	1.00	1.00
Natural gas	584 (15.1)	74 (12.7)	1.63 (1.04–2.64)*	1.75 (1.08–2.91)*	1.57 (0.97–2.62)	1.45 (0.89–2.43)
Kerosene	2835 (73.2)	379 (13.4)	1.74 (1.18–2.67)*	1.94 (1.29–3.04)*	1.75 (1.15–2.74)*	1.58 (1.03–2.49)*
Other	124 (3.2)	16 (12.9)	1.67 (0.85–3.18)	1.90 (0.94–3.74)	1.78 (0.88–3.50)	1.62 (0.80–3.20)
Flue status (except electric heaters)						
Flued	2775 (78.3)	343 (12.4)	1.00	1.00	1.00	1.00
Unflued	768 (21.7)	126 (16.4)	1.39 (1.11–1.73)*	1.36 (1.08–1.71)*	1.32 (1.04–1.65)*	1.29 (1.02–1.63)*
Ventilation						
Yes	2533 (65.4)	302 (11.9)	1.00	1.00	1.00	1.00
No	1341 (34.6)	194 (14.5)	1.25 (1.03–1.52)*	1.24 (1.01–1.52)*	1.22 (1.00–1.50)	1.19 (0.97–1.45)

Ever asthma symptoms						
Use of heating fuels						
Electricity	331 (8.5)	91 (27.5)	1.00	1.00	1.00	1.00
Natural gas	584 (15.1)	187 (32.0)	1.24 (0.92–1.68)	1.17 (0.85–1.62)	1.10 (0.79–1.53)	1.07 (0.77–1.49)
Kerosene	2835 (73.2)	883 (31.2)	1.19 (0.93–1.54)	1.20 (0.92–1.58)	1.12 (0.85–1.48)	1.08 (0.82–1.44)
Other	124 (3.2)	36 (29.0)	1.08 (0.68–1.69)	1.09 (0.67–1.75)	1.03 (0.63–1.67)	1.00 (0.61–1.62)
Flue status (except electric heaters)						
Flued	2775 (78.3)	850 (30.6)	1.00	1.00	1.00	1.00
Unflued	768 (21.7)	256 (33.3)	1.13 (0.95–1.34)	1.09 (0.91–1.30)	1.06 (0.88–1.26)	1.05 (0.88–1.26)
Ventilation						
Yes	2533 (65.4)	755 (29.8)	1.00	1.00	1.00	1.00
No	1341 (34.6)	442 (33.0)	1.16 (1.00–1.33)*	1.15 (0.99–1.34)	1.14 (0.98–1.33)	1.13 (0.97–1.32)

Values are expressed as number (percentage). OR, odds ratio; CI, confidence interval.

*P < 0.05.

^a^Adjusted for sex, school grade, parental history of allergies, and schools.

^b^Adjusted for sex, school grade, parental history of allergies, schools, residence within 200 meters of a main road, presence of wall-to-wall carpeting in the home, and presence of smoker in the home.

^c^Adjusted for sex, school grade, parental history of allergies, schools, residence within 200 meters of a main road, presence of wall-to-wall carpeting in the home, presence of a smoker in the home, and presence of indoor dampness.

Table 4 shows associations of asthma symptoms with the combination of heating system and ventilation. After adjusting for potential confounding factors in Model 3, use of flued heaters without ventilation (OR = 1.62; 95% CI, 1.03–2.64) was significantly associated with current asthma symptoms. Use of unflued heaters was also significantly associated with current asthma symptoms in the presence (OR = 1.77; 95% CI, 1.09–2.95) and absence of ventilation (OR = 2.23; 95% CI, 1.31–3.85), with use of electric heaters as reference. Only use of unflued heaters without ventilation was significantly associated with ever asthma symptoms (OR = 1.47; 95% CI, 1.01–2.14).

**Table 4.** Multivariate analyses of the association of heating systems/mechanical ventilation (ventilation) status with asthma symptoms

Variable	*n* (%)	Asthma symptoms *n* (%)	Crude OR (95% CI)	Model 1 OR^a^ (95% CI)	Model 2 OR^b^ (95% CI)	Model 3 OR^c^ (95% CI)
Current asthma symptoms						
Electric heaters	331 (8.6)	27 (8.2)	1.00	1.00	1.00	1.00
Flued heaters with ventilation	1782 (46.0)	207 (11.6)	1.48 (0.99–2.30)	1.66 (1.09–2.63)*	1.53 (0.99–2.43)	1.41 (0.91–2.25)
Flued heaters without ventilation	993 (25.6)	136 (13.7)	1.79 (1.18–2.81)*	2.00 (1.28–3.21)*	1.80 (1.15–2.91)*	1.62 (1.03–2.64)*
Unflued heaters with ventilation	501 (12.9)	76 (15.2)	2.01 (1.28–3.25)*	2.24 (1.40–3.69)*	1.96 (1.22–3.25)*	1.77 (1.09–2.95)*
Unflued heaters without ventilation	267 (6.9)	50 (18.7)	2.59 (1.59–4.32)*	2.79 (1.66–4.76)*	2.48 (1.47–4.26)*	2.23 (1.31–3.85)*

Ever asthma symptoms						
Electric heaters	331 (8.6)	91 (27.5)	1.00	1.00	1.00	1.00
Flued heaters with ventilation	1782 (46.0)	536 (30.1)	1.13 (0.88–1.48)	1.14 (0.86–1.51)	1.09 (0.83–1.46)	1.06 (0.80–1.42)
Flued heaters without ventilation	993 (25.6)	314 (31.6)	1.22 (0.93–1.61)	1.23 (0.91–1.65)	1.17 (0.87–1.59)	1.13 (0.83–1.54)
Unflued heaters with ventilation	501 (12.9)	151 (30.1)	1.14 (0.84–1.55)	1.11 (0.80–1.54)	1.06 (0.76–1.47)	1.02 (0.73–1.43)
Unflued heaters without ventilation	267 (6.9)	105 (39.3)	1.71 (1.21–2.42)*	1.61 (1.12–2.34)*	1.53 (1.05–2.21)*	1.47 (1.01–2.14)*

Values are expressed as number (percentage). OR, odds ratio; CI, confidence interval.

*P < 0.05.

^a^Adjusted for sex, school grade, parental history of allergies, and schools.

^b^Adjusted for sex, school grade, parental history of allergies, schools, residence within 200 meters of a main road, presence of wall-to-wall carpeting in the home, and presence of a smoker in the home.

^c^Adjusted for sex, school grade, parental history of allergies, schools, residence within 200 meters of a main road, presence of wall-to-wall carpeting in the home, presence of a smoker in the home, and presence of indoor dampness.

Table 5 shows the results stratified by indoor dampness. Presence of dampness was associated with a higher prevalence of current asthma symptoms among households using electric heaters (OR = 1.83; 95% CI, 0.73–4.31), although the result was not significant. In homes without indoor dampness, use of unflued heaters was significantly associated with current asthma symptoms in the presence (OR = 2.42; 95% CI, 1.22–4.85) and absence of ventilation (OR = 3.26; 95% CI, 1.46–7.18), as compared with use of electric heaters. Likewise, in homes with indoor dampness, use of flued or unflued heaters in the presence (flued: OR = 2.02; 95% CI, 1.20–3.59; unflued: OR = 2.35; 95% CI, 1.31–4.35) and absence (flued: OR = 2.47; 95% CI, 1.45–4.42; unflued: OR = 2.89; 95% CI, 1.55–5.57) of mechanical ventilation was significantly associated with current asthma symptoms. Only use of unflued heaters without ventilation in the presence of dampness was significantly associated with ever asthma symptoms (OR = 1.79; 95% CI, 1.16–2.27).

**Table 5.** Multivariate analyses of the association of heating system/mechanical ventilation (ventilation) status with asthma, stratified by indoor dampness status

Heating systems and ventilation	*n* (%)	Asthma symptoms *n* (%)	OR^a^ (95% CI)
Current asthma symptoms			
Absence of indoor dampness			
Electric heaters	258 (16.5)	18 (7.0)	1.00
Flued heaters with ventilation	744 (47.7)	74 (10.0)	1.57 (0.91–2.83)
Flued heaters without ventilation	318 (20.4)	29 (9.1)	1.51 (0.80–2.92)
Unflued heaters with ventilation	160 (10.3)	23 (14.4)	2.42 (1.22–4.85)*
Unflued heaters without ventilation	79 (5.1)	14 (17.7)	3.26 (1.46–7.18)*
Presence of indoor dampness			
Electric heaters	73 (3.2)	9 (12.3)	1.83 (0.73–4.31)
Flued heaters with ventilation	1038 (44.8)	133 (12.8)	2.02 (1.20–3.59)*
Flued heaters without ventilation	675 (29.2)	107 (15.8)	2.47 (1.45–4.42)*
Unflued heaters with ventilation	341 (14.7)	53 (15.5)	2.35 (1.31–4.35)*
Unflued heaters without ventilation	188 (8.1)	36 (19.2)	2.89 (1.55–5.57)*

Ever asthma symptoms			
Absence of indoor dampness			
Electric heaters	258 (16.5)	68 (26.4)	1.00
Flued heaters with ventilation	744 (47.7)	220 (29.6)	1.16 (0.82–1.64)
Flued heaters without ventilation	318 (20.4)	78 (24.5)	0.92 (0.61–1.37)
Unflued heaters with ventilation	160 (10.3)	52 (32.5)	1.41 (0.89–2.24)
Unflued heaters without ventilation	79 (5.1)	25 (31.6)	1.24 (0.68–2.21)
Presence of indoor dampness			
Electric heaters	73 (3.2)	23 (31.5)	1.22 (0.67–2.20)
Flued heaters with ventilation	1038 (44.8)	316 (30.4)	1.16 (0.83–1.63)
Flued heaters without ventilation	675 (29.2)	236 (35.0)	1.40 (0.99–2.01)
Unflued heaters with ventilation	341 (14.7)	99 (29.0)	1.01 (0.68–1.50)
Unflued heaters without ventilation	188 (8.1)	80 (42.6)	1.79 (1.16–2.27)*

Values are expressed as number (percentage). OR, odds ratio; CI, confidence interval.

*P < 0.05.

^a^Adjusted for sex, school grade, parental history of allergies, schools, residence within 200 meters of a main road, presence of wall-to-wall carpeting in the home, and presence of a smoker in the home.

## DISCUSSION

In this study, as compared with use of electric heaters, use of unflued fume-emitting gas or kerosene heaters and use of fume-emitting gas or kerosene heaters without ventilation were associated with current asthma symptoms in children. The association of use of unflued heaters in the absence of ventilation with current asthma symptoms was stronger than that for unflued heaters in the presence of ventilation. In addition, the association of use of unflued heaters in the absence of ventilation with current asthma symptoms persisted regardless of indoor dampness. In contrast, these associations were not found in children who had ever asthma symptoms. The home environment may have changed as the child grew up, or asthma symptoms may have occurred in the early years of their lives. It is therefore possible that current asthma symptoms is associated with current home environment.

This study was conducted in public primary schools in Sapporo. Questionnaires were distributed to all children in these schools, and the response rate was 69.5%. After excluding questionnaires with missing information, data from 3874 children (60.6%) were analyzed. As compared with the data for the 4445 children who responded, the data for sex, school grade, and parental history of allergies did not differ between the children with and without asthma symptoms among the 3874 children analyzed. Therefore, our results are generalizable to elementary school children living in northern Japan.

Earlier data showed that 88.3% of households used kerosene or gas heaters and that houses in Sapporo are typically airtight.^27^ Because our results suggest that heating without adequate ventilation has serious adverse effects on asthma symptoms, our results might aid in preventing asthma in other areas of the world with similarly airtight homes.

The World Health Organization reported that dampness and mold are the most important indoor environmental factors in increasing the prevalence of respiratory symptoms, allergies, and asthma.^28^ Previous studies have indicated that dampness is also a strong risk factor for asthma.^11^^–^^13^ Dampness is associated with use of fume-emitting heaters without a flue (unflued heaters) and absence of ventilation in the home.^28^ Therefore, it is possible that the association of unflued heaters or absence of ventilation with asthma symptoms is mediated by dampness. To confirm this, we conducted an analysis stratified by dampness status. We found that, among households using electric heaters, presence of dampness was associated with a nonsignificant increase in current asthma symptoms, as compared with absence of dampness. We also found that, regardless of indoor dampness, use of unflued heaters, with or without ventilation, was associated with current asthma symptoms. Thus, although dampness could be a mediating factor, air pollution from fume-emitting heaters may also explain the association between use of unflued heaters with or without ventilation and current asthma symptoms.

Several cross-sectional studies found that use of gas^17^^,^^29^ and kerosene^18^ heating were risk factors for asthma in children. However, another study reported no significant association between use of such heaters and asthma symptoms.^19^ Our results showed that only kerosene heaters had an effect on asthma; the effect of gas heaters was not statistically significant after adjusting for potential confounding factors. Because unflued heaters may have an important role in asthma, we combined heating fuel with flue status to assess the effects on asthma. As compared with the use of electric heaters, use of unflued heaters increased asthma risk. Two randomized controlled trials suggested that replacement of unflued gas heaters with flued gas heaters decreased asthma symptoms in children.^20^^,^^21^ In this study, we found that use of unflued heaters was associated with current asthma symptoms, which is consistent with earlier findings.

Gas and kerosene fuel stoves emit nitrogen dioxide (NO_2_), sulfur dioxide (SO_2_), and particulate matter (PM),^30^^,^^31^ which are associated with asthma symptoms in children.^32^ NO_2_ is a by-product of high-temperature combustion from stoves and was found to exacerbate wheezing in children with asthma.^33^ Inhaled NO_2_ increased the likelihood of initial sensitization to house-dust mites and has a role in the development of atopic asthma.^34^ In another study, inhaled NO_2_ increased lung resistance, respiratory rate, and minute ventilation.^35^ Furthermore, NO_2_ and SO_2_ can cause wheezing and breathlessness^36^ and enhance airway response to inhaled allergens.^37^ Increased indoor PM concentrations were associated with asthma exacerbation in children.^38^ In mice, PM exposure increased the pathophysiologic features of asthma by activating lymphocyte-dependent pathways.^39^ The biological effects of PM from gas stoves are thought to be due to oxidative stress, which results in cell signaling, transcription factor activation, and mediator release in the respiratory tract, eventually culminating in inflammation.^40^ Therefore, use of unflued fume-emitting heaters may be associated with current asthma symptoms.

We also assessed the combined effect of heating systems and ventilation on asthma symptoms. Use of unflued heaters in the absence of ventilation increased asthma risk, as compared with use of unflued heaters in the presence of ventilation. Previous studies found that inadequate ventilation in homes that use unflued heaters increased asthma risk.^23^^,^^31^^,^^41^ Improved ventilation decreased mean indoor NO_2_ levels produced by gas stoves^31^^,^^41^ and can be a useful way to decrease indoor air pollution.^23^ Therefore, to decrease asthma symptoms in children, houses that use unflued fume-emitting heaters should be ventilated.

This study had several limitations. First, causal relationships could not be ascertained, due to the cross-sectional nature of the study. Furthermore, before this study, parents might have changed their heating system or ventilation status due to their child’s asthma symptoms. Such actions would diminish the association between use of unflued heaters without ventilation and asthma symptoms. Indeed, for ever asthma symptoms, a significant (but weak) association was found only for use of unflued heaters without ventilation. Second, asthma symptoms were not physician-diagnosed; however, we evaluated patients using the ISAAC questionnaire, which is used worldwide.^26^ Therefore, the results should be considered reliable. Third, we did not measure other major risk factors for asthma, such as socioeconomic status^42^ and presence of house-dust mite allergens.^34^^,^^43^ These factors may have been confounders. However, we controlled extensively for other potential confounders and found strong associations of use of unflued heaters and absence of mechanical ventilation with current asthma symptoms. Moreover, the risk factors for asthma, such as use of carpeting^9^ and presence of tobacco smoke,^8^ indoor dampness, and mold,^11^^–^^13^ were consistent with those examined previously and were controlled for in this study. Therefore, it is likely that our results are representative and that the associations are valid. Fourth, information on heating systems and ventilation was obtained solely from parents; thus, misclassification might have occurred. However, it would have been quite simple for parents to identify the type of heating system they had and whether they had (mechanical) ventilation. Furthermore, they would have been able to recognize flued or unflued heaters by the presence or absence of vent pipes to the outside. Fifth, children spend a lot of time in elementary school. However, we did not have detailed information on the schools. The prevalence of asthma symptoms in the 12 schools was different; nevertheless, the present results did not change after adjusting for school in the multivariate analyses. Sixth, we had no information on the time children spent in the home and how they used the heater during the daytime and nighttime. Some children may not have spent a long time at home or may have used heaters for only a short time. If we had assessed duration and frequency of heater use in the home, the results might have been even clearer. We are planning to assess duration and frequency of heater use in the home and the duration spent at home in a future study.

In conclusion, as compared with use of electric heaters, use of kerosene heaters, but not use of gas heaters, was significantly associated with current asthma symptoms in children. However, when we considered use of flues and presence of ventilation, there were significant differences related to use of fume-emitting heaters such as kerosene and gas heaters. As compared with use of electric heaters, use of unflued fume-emitting kerosene or gas heaters was associated with current asthma symptoms, and this association was stronger among participants who lived in houses with unflued kerosene or gas heaters in the absence of ventilation. In addition, this association of unflued heaters in the absence of ventilation persisted regardless of the presence of indoor dampness. Ever asthma symptoms was also associated with use of unflued heaters without ventilation. Therefore, in northern cities like Sapporo, particular attention needs to be paid to the use of fuel heaters, especially those that have no flue or ventilation, due to their possible association with asthma symptoms in children.

## ONLINE ONLY MATERIALS

Abstract in Japanese.
