# Supplementary material for: Association of Mechanical Ventilation and Flue Use in Heaters With Asthma Symptoms in Japanese Schoolchildren: A Cross-Sectional Study in Sapporo, Japan
Source: J Epidemiol. 2014 May 5;24(3):230–8. doi: 10.2188/jea.JE20130135 (PMC4000771; doi:10.2188/jea.JE20130135)
Supplement: Abstract in Japanese. [file je-24-230-s001.pdf]

札幌市児童の喘息と自宅の暖房器具の排気管および機械換気との関連 － 横断研究

叢石[1]、荒木敦子[2]、鵜川重和[1]、アイツバマイゆふ[1]、多島秀司[2]、金澤文子[1]、湯浅資之[1]、玉腰暁子[1]、岸玲子[2]

[1]北海道大学大学院医学研究科公衆衛生学分野、[2]北海道大学環境健康科学研究教育センター

【背景】暖房器具の種類と児童の喘息との関連が報告されている。しかし、これまでに児童の喘息と暖房器具の排気管、および機械換気との関連を評価した研究はなかった。

【方法】札幌市で小学校 1 年生から 6 年生（6–12 歳）を対象に横断研究を行った。2008 年 11 月から 2009 年 1 月にかけて、保護者に住宅環境および児童の喘息症状に関する調査票の記入を依頼した。

【結果】6393 名の児童のうち、4445 人から調査票を回収し（回収率 69.5%)、欠損データを除外した 3874 人のデータを分析した。現在喘息症状がある児童は 12.8%、今までに喘息症状がある児童は 30.9%であった。電気暖房と比較し、ガス、石油などの暖房で排気管あり・機換換気なしの場合（OR = 1.62; 95% CI, 1.03-2.64)、排気管なし・機械換気ありの場合（OR = 1.77; 95% CI, 1.09-2.95)、排気管なし・機械換気なしの場合（OR = 2.23; 95% CI, 1.31 -3.85）は、いずれも現在の喘息症状のオッズ比が有意に高かった。ダンプネスの有無に関わらず、排気管なしの暖房を利用している場合は機械換気がある場合とない場合のいずれも現在の喘息症状と有意に関連していた。

【結論】ダンプネスがあってもなくても、排気管なしの暖房器具を使うことは現在の喘息症状と関連していた。特に、機械換気がある場合と比べ、機械換気がない場合は現在の喘息症状のオッズ比が有意に高かった。一方、今までに喘息症状があることは、排気管がない暖房器具を機械換気なしで使用する場合とのみ関連していた。したがって、ガス、石油等の暖房器具に排気管が取り付けられない場合、および機械換気がない場合には、児童の喘息に関連する可能性があることに十分注意することが必要である。

キーワード：喘息、暖房器具、機械換気、児童、室内空気質
